# Lung cancer and the eroded self: identity challenges and mental well-being – a narrative review

**DOI:** 10.1097/MS9.0000000000004190

**Published:** 2025-10-28

**Authors:** Emmnauel Ifeanyi Obeagu, Aakib Rahman Parray

**Affiliations:** a Department of Biomedical and Laboratory Science, Africa University, Zimbabwe; b Department of Psychology, Akal University, Punjab, India

**Keywords:** identity disruption, lung cancer, mental health, psychological resilience, stigma

## Abstract

Lung cancer remains a leading cause of cancer-related mortality worldwide, but its psychological burden often receives less attention than its physical toll. Unlike other cancers, lung cancer carries a significant stigma, frequently linked to smoking and lifestyle choices. This stigma contributes to internalized guilt and shame, which can profoundly disrupt a patient’s sense of self. The diagnosis often triggers an identity crisis, where individuals struggle to reconcile their pre-illness identity with their new reality, leading to heightened emotional distress, self-stigmatization, and social withdrawal. As the disease progresses, patients frequently experience role loss in their personal and professional lives due to physical limitations, altered appearance, and reduced functional capacity. These disruptions contribute to a fractured sense of identity and diminished self-worth. The unique psychosocial landscape of lung cancer often includes higher rates of anxiety, depression, and suicidal ideation compared to other cancer populations. Unfortunately, identity-based psychological suffering is rarely addressed in standard oncology care, leaving many patients without the support they need to navigate these challenges. Emerging interventions – such as narrative therapy, dignity therapy, and acceptance-based approaches – offer promising avenues to help patients reconstruct their identity, reclaim meaning, and improve psychological resilience. Addressing the erosion of self in lung cancer patients is critical for delivering holistic, patient-centered care. By confronting stigma, validating patients’ experiences, and integrating mental health support into routine oncology practice, we can foster healing not only of the body but of the self.

## Introduction

Lung cancer is not only one of the most lethal malignancies worldwide, but also one of the most psychologically taxing[1],[2]. It is often associated with a heavy emotional burden that extends beyond the clinical manifestations of the disease. Unlike many other cancers, lung cancer frequently carries a societal stigma that profoundly impacts the mental and emotional well-being of those diagnosed[3]. Patients commonly internalize blame for their condition, particularly when their history includes tobacco use, creating a unique psychosocial environment characterized by shame, isolation, and identity disruption^[3,4]^. Identity – the sense of who one is and how one fits into the world – is deeply intertwined with health, bodily integrity, and social roles. A cancer diagnosis, particularly one as aggressive and stigmatized as lung cancer, can fracture this identity^[2,5,6]^. Suddenly, patients are forced to reassess their sense of self, as roles they once held – such as worker, caregiver, partner, or provider – become compromised by illness[7]. The transition from being healthy to being “a cancer patient” can be abrupt and emotionally destabilizing, stripping individuals of the continuity that anchors their personal narrative.

Stigma intensifies this disruption. Public assumptions that lung cancer is self-inflicted due to smoking contribute to a culture of blame, often diminishing the empathy afforded to those with other cancers[8]. Even nonsmokers with lung cancer report experiencing judgment and diminished support[9]. This atmosphere of blame can cause individuals to withdraw socially, exacerbating feelings of alienation and diminishing their perceived worth^[10,11]^. Such stigma, both external and internalized, undermines psychological resilience and impedes efforts to adapt positively to the diagnosis. The transformation of the body also plays a key role in identity erosion. Treatments such as chemotherapy, radiation, and surgery may result in disfigurement, fatigue, hair loss, and respiratory complications, all of which alter how patients perceive themselves^[12,13]^. This can be particularly distressing when the physical changes clash with one’s precancer identity – especially in younger patients, those with active lifestyles, or individuals for whom physicality played a central role in self-definition. The resulting dissonance between body and identity deepens the psychological toll^[14,15]^. Moreover, lung cancer often progresses rapidly, leaving little time for psychological adjustment^[16,17]^. Patients may find themselves in a constant state of crisis – navigating diagnosis, treatment, and existential threats in a compressed timeline^[17,18]^. In this context, the psychological work of maintaining or reconstructing a coherent self-image becomes increasingly difficult. The emotional demands of coping with uncertainty, mortality, and ongoing medical intervention frequently lead to heightened levels of anxiety, depression, and despair.

## Aim

This narrative review aims to explore the complex psychological challenges faced by individuals diagnosed with lung cancer, focusing on how the disease and its associated stigma disrupt personal identity and mental well-being.

## Narrative review methods

This narrative review was conducted to synthesize existing literature on the psychological aspects of lung cancer, with a particular focus on identity disruption and mental well-being. A comprehensive search was performed across multiple electronic databases including PubMed, PsycINFO, Scopus, and Google Scholar. The search terms included combinations of keywords such as “lung cancer,” “psychological impact,” “identity,” “stigma,” “mental health,” “depression,” “anxiety,” and “psychosocial interventions.” Articles published in English between 2000 and 2025 were considered to capture recent advances in psycho-oncology. Both qualitative and quantitative studies, systematic reviews, and relevant theoretical papers were included to provide a broad and in-depth understanding of the topic. Studies focusing specifically on lung cancer patients’ experiences, mental health outcomes, and psychosocial care were prioritized. Selected articles were screened by title and abstract for relevance, followed by full-text review. Data were extracted and thematically analyzed to identify key psychological challenges, the impact of stigma and identity disruption, mental health outcomes, and effective therapeutic approaches. This narrative synthesis aimed to integrate diverse findings into a coherent framework to inform clinical practice and future research directions.

## The psychological toll of diagnosis of lung cancer

The moment of lung cancer diagnosis often triggers a profound psychological crisis[19]. For many individuals, the announcement is not merely a medical revelation – it is a rupture in their personal narrative and a shattering of life’s perceived continuity. Unlike diagnoses of less stigmatized cancers[20], lung cancer is frequently accompanied by a complex cocktail of emotions: fear of death, grief over the life that may never be lived, shame stemming from perceived culpability[21], and confusion over the next steps in care^[3,22]^. These emotions can be immediate and overwhelming, often preceding any physical symptoms or treatment-related distress. A unique and painful aspect of lung cancer diagnosis is the automatic association with smoking, which invites both self-blame and social judgment[8]. Even patients who have never smoked or quit years ago may internalize this blame, questioning their past choices and grappling with feelings of guilt[23]. This differs from the emotional response seen in other cancers where lifestyle factors are less scrutinized[2]. The internal dialog – “Did I do this to myself?” – often creates a psychological burden that exacerbates anxiety and depression^[24,25]^. Patients may struggle not only with the reality of a life-threatening illness but also with moral injury and diminished self-worth. Beyond stigma, the diagnosis marks the beginning of a personal identity crisis[26]. Patients suddenly find themselves transitioning from an autonomous individual to a “cancer patient” under constant medical surveillance. This role shift can lead to a profound sense of loss – of independence, purpose, control, and normalcy^[27]^. Many patients report a narrowing of identity, in which the diagnosis eclipses their previous sense of self, professional identity, and future aspirations[28]. The sudden urgency of medical appointments, discussions of prognosis, and contemplation of mortality leaves little room for emotional processing, often resulting in acute psychological distress that persists well into treatment[29] (see Table 1).

**HIGHLIGHTS**

- Lung cancer disrupts personal identity, often leading to a fractured sense of self.
- Patients experience stigma, especially if linked to smoking.
- Mental health struggles include anxiety, depression, and isolation.
- Physical decline undermines autonomy and self-image.
- Supportive care must address both emotional and existential dimensions.

**Table 1:** The psychological toll of diagnosis of lung cancer

Psychological aspect	Description	Clinical implications
Emotionalshock	Initial intense feelings of fear, grief, confusion, and disbelief immediately following diagnosis.	Requires immediate emotional support and clear communication.
Fear of mortality	Heightened awareness of death and uncertainty about life expectancy.	May lead to anxiety and existential distress needing psychosocial intervention.
Self-blame and guilt	Internalized responsibility linked to smoking history or perceived lifestyle choices.	Can exacerbate depression and delay help-seeking behaviors.
Social stigma	Experience of judgment and blame from others due to lung cancer’s association with smoking.	Calls for stigma-reduction education among healthcare providers and communities.
Identity disruption	Sudden transition from healthy individual to “cancer patient” with loss of normalcy and control.	Necessitates support for identity reconstruction and coping.
Psychological distress	High levels of anxiety, depression, and hopelessness soon after diagnosis.	Screening and timely mental health referrals are essential.

## Disruption of identity and roles in lung cancer patients

The diagnosis and progression of lung cancer often precipitate a significant disruption in personal identity and social roles[30]. For many patients, cancer is not merely a health crisis but an event that radically alters how they see themselves and how they are seen by others. The transformation from a previously healthy and functional individual into someone who is dependent on medical systems and caregivers can be deeply disorienting. This identity shift is particularly jarring in lung cancer due to the rapid disease trajectory, visible physical decline, and societal stigma that often accompanies the illness^[31,32]^. One of the most profound identity disruptions occurs in the domain of occupational and familial roles^[6,33]^. Patients who were once breadwinners, caregivers, or active community members may suddenly find themselves unable to meet these expectations due to physical limitations such as fatigue, dyspnea, and pain. This role loss can lead to a diminished sense of purpose and worth[34]. Men, in particular, may struggle with traditional masculine identities tied to productivity and stoicism,^[35,36]^ while women may experience distress over their inability to fulfill nurturing or domestic responsibilities[37]. The inability to contribute meaningfully within the family or workplace can create feelings of helplessness and irrelevance. Moreover, lung cancer alters how patients relate to their own bodies and time[38]. Changes in physical appearance due to treatment – such as hair loss, weight changes, or surgical scars – can distort body image and further estrange individuals from their pre-illness identity^[15,39,40].^ The future, once a canvas for long-term planning and aspiration, becomes compressed into cycles of treatment and survival. This temporal disruption complicates the process of identity continuity, as patients are forced to revise life goals, abandon previous dreams, and grapple with uncertainty. In response, many patients report a sense of existential dislocation, describing themselves as “no longer the person they used to be.” Reconstructing a stable sense of self under these conditions requires emotional support, narrative integration, and, often, professional psychosocial intervention[41].

## The burden of stigma and self-blame in lung cancer

Stigma remains one of the most psychologically corrosive aspects of living with lung cancer. Unlike many other forms of cancer, lung cancer is often regarded by the public as a self-inflicted disease due to its historical association with smoking^[42,43]^. This perception fosters a unique and pervasive form of blame that patients must navigate alongside their physical illness. Even when a patient’s cancer is unrelated to tobacco use – as is increasingly the case among non-smokers, particularly women – societal narratives continue to cast suspicion, leading to misinformed assumptions and diminished empathy from healthcare providers, caregivers, and peers^[44,45]^. This external stigma frequently becomes internalized, resulting in profound self-blame. Patients may ruminate over past behaviors, lifestyle choices, or environmental exposures, reinforcing the belief that their suffering is deserved or preventable. Such cognitive patterns contribute to a cycle of shame and guilt that can obstruct emotional healing and lead to psychological withdrawal[46]. Self-blame has been directly linked to higher levels of anxiety, depression, and even suicidal ideation in lung cancer patients[47]. It also discourages open communication with clinicians, potentially reducing access to psychosocial support or symptom management strategies. Moreover, stigma impacts not only individual mental health but also patient advocacy, funding, and research attention[31]. Lung cancer patients often report feeling like second-class citizens in the cancer community – less likely to be celebrated, supported, or invited to share their stories compared to individuals with other malignancies[48]. This invisibility compounds emotional isolation and fuels a sense of unworthiness. In some cases, patients may delay seeking medical help due to fear of judgment, leading to more advanced disease at presentation[49]. Addressing the burden of stigma requires a multi-level response, including public education to dismantle harmful stereotypes, clinical empathy training, and robust mental health support integrated into oncology care.

## Mental health outcomes in lung cancer

The psychological impact of lung cancer is profound, with mental health outcomes among the most severe across all cancer types. Numerous studies have documented high rates of psychiatric comorbidities in individuals diagnosed with lung cancer, including depression, anxiety disorders, adjustment disorders, and post-traumatic stress symptoms^[50,51]^. These outcomes are influenced by a constellation of factors – disease severity, physical symptoms, stigma, perceived prognosis, and disrupted identity – creating a perfect storm for psychological distress[52]. Notably, depressive symptoms in lung cancer are not only more prevalent but also more persistent and severe compared to those in patients with other cancers. Depression in lung cancer patients is often underdiagnosed and undertreated, yet it has significant consequences for both quality of life and clinical outcomes. Depression has been linked to poorer treatment adherence, diminished physical functioning, reduced immune response, and even shortened survival^[53,54]^. Moreover, anxiety—often driven by fear of disease progression, treatment side effects, or death – can exacerbate physical symptoms such as dyspnea, further compounding distress.

Patients may experience anticipatory grief and existential despair as they struggle with the threat of mortality and the progressive loss of autonomy and control. Suicidality is another critical mental health concern in lung cancer[55]. Research shows that individuals with lung cancer have a significantly higher risk of suicide compared to the general population and to those with other types of cancer^[56,57]^. This alarming statistic reflects the depth of psychological suffering many patients endure, often in silence. Contributing factors include unmanaged pain, inadequate psychosocial support, and the cumulative effects of isolation and stigma. These findings underscore the urgency of integrating mental health services into oncology care. Routine screening for psychological distress, timely referrals to psycho-oncology specialists, and access to palliative mental health interventions are essential to safeguarding the emotional well-being of patients navigating the lung cancer journey.

## Pathways to identity reconstruction in lung cancer

Despite the profound challenges to selfhood posed by lung cancer, many patients engage in processes of identity reconstruction that help restore a coherent sense of self and improve mental well-being. This reconstruction often involves reinterpreting the illness experience in ways that preserve personal dignity, meaning, and continuity[58]. Psychosocial interventions aimed at facilitating narrative integration and acceptance have demonstrated promise in supporting patients through these transformative journeys. One key approach is meaning-centered psychotherapy, which helps patients explore and affirm sources of meaning beyond their illness, such as relationships, spirituality, or personal values^[59,60]^. By fostering a broader sense of purpose, this therapy encourages patients to view their cancer diagnosis as part of a larger life story rather than an all-defining catastrophe. Similarly, dignity therapy invites patients to reflect on their life achievements, important memories, and hopes for legacy, thereby strengthening identity and alleviating existential distress^[61,62]^. These interventions can mitigate feelings of isolation, helplessness, and worthlessness that often accompany lung cancer.

Complementary to formal therapies, social support plays a critical role in identity reconstruction. Support groups and peer mentorship offer spaces where patients share experiences, normalize their feelings, and renegotiate social roles in a community of understanding^[63–65]^. Family involvement is also crucial, as adaptive family dynamics can reinforce patients’ valued identities and provide emotional scaffolding^[66,67].^ Lastly, mindfulness-based strategies and acceptance and commitment therapy (ACT) enable patients to develop psychological flexibility, helping them accept their illness and focus on living in accordance with chosen values^[68,69]^. Together, these pathways offer a multifaceted toolkit for lung cancer patients striving to reclaim a stable and meaningful sense of self amidst the turbulence of disease[70].

## Clinical implications

Recognizing the profound psychological and identity-related challenges faced by lung cancer patients has important implications for clinical practice. Integrating routine mental health screening into oncology care is essential to identify patients experiencing distress, depression, anxiety, or stigma-related isolation early in the disease trajectory. Simple, validated tools such as the Hospital Anxiety and Depression Scale (HADS) or the Distress Thermometer can be employed to efficiently flag patients in need of further psychological assessment and support. Beyond screening, multidisciplinary care models that incorporate psycho-oncology professionals, social workers, and chaplains can provide comprehensive psychosocial support tailored to individual patient needs. Mental health interventions should address not only symptom management but also identity reconstruction, stigma reduction, and meaning-making to holistically improve patient well-being. Training oncology teams in empathetic communication and stigma awareness can enhance patient–provider relationships and encourage open discussions about emotional and existential concerns. Furthermore, involving family members and caregivers in supportive care planning helps to maintain patients’ valued roles and social connections, which are vital for sustaining identity and resilience. Health systems should also prioritize accessible, culturally sensitive psychosocial services, including telehealth options, to overcome barriers such as physical limitations and stigma that may deter patients from seeking help. Ultimately, embedding mental healthcare within lung cancer treatment pathways not only improves quality of life but may also positively influence clinical outcomes by fostering treatment adherence and overall coping capacity.

## Conclusion

Lung cancer profoundly challenges patients’ sense of identity, exacerbated by stigma, role disruption, and the physical toll of the disease. These factors intertwine to erode the self and contribute to significant mental health burdens, including depression, anxiety, and existential distress. Yet, amidst these challenges, pathways to identity reconstruction offer hope, highlighting the critical role of psychosocial support in helping patients reclaim a coherent and meaningful sense of self. Addressing the psychological dimensions of lung cancer must become a central component of comprehensive care. Early identification of distress, stigma reduction efforts, and tailored interventions that focus on restoring dignity and meaning can significantly enhance patients’ mental well-being and quality of life. Moreover, integrating family support and fostering empathetic healthcare environments can mitigate the isolating effects of lung cancer and promote resilience.

## Data Availability

Not applicable as this a narrative review.

## Research registration unique identifying number (UIN)

Not applicable as this a narrative review.
